# Is the Neutrophil-to-Lymphocyte Ratio a Predictive Factor of Pathological Complete Response in Egyptian Breast Cancer Patients Treated with Neoadjuvant Chemotherapy?

**DOI:** 10.3390/medicina61020327

**Published:** 2025-02-13

**Authors:** Naglaa F. Ebaid, Khaled S. Abdelkawy, Amira S. A. Said, Mohamad M. Al-Ahmad, Mohamed A. Shehata, Heba F. Salem, Raghda R. S. Hussein

**Affiliations:** 1 Clinical Pharmacy Department, Faculty of Pharmacy, Menoufia University, Menoufia 32511, Egypt; naglafathy81@gmail.com; 2 Clinical Pharmacy Department, Faculty of Pharmacy, Kafrelsheikh University, Kafr El Sheikh 33516, Egypt; ksibrahi@ualberta.ca; 3 Department of Clinical Pharmacy, College of Pharmacy, Al Ain University, Al Ain P.O. Box 64141, United Arab Emirates; amira.ahmed@aau.ac.ae (A.S.A.S.); mohammad.alahmad@aau.ac.ae (M.M.A.-A.); 4 Clinical Oncology and Nuclear Medicine Department, Faculty of Medicine, Menoufia University, Menofia 32511, Egypt; mohaboulfetouh@yahoo.com; 5 Pharmaceutics and Industrial Pharmacy Department, Beni-Suef University, Beni-Suef 62574, Egypt; heba_salem2004@yahoo.co.uk; 6 Clinical Pharmacy Department, Faculty of Pharmacy, Beni-Suef University, Beni-Suef 62574, Egypt

**Keywords:** breast cancer, neoadjuvant chemotherapy, pathological complete response, neutrophil-to-lymphocyte ratio

## Abstract

*Background and Objectives:* The role of the neutrophil-to-lymphocyte ratio (NLR) as a predictor of response in breast cancers after neoadjuvant chemotherapy is controversial. This study aims to explore the relationship of NLR with pathological complete response (pCR) in a cohort of Egyptian breast cancer patients who received neoadjuvant chemotherapy. *Materials and Methods:* Forty-six breast cancer females received preoperative neoadjuvant chemotherapy and then underwent surgery. All resected tumors were evaluated to determine the pathologic effect of the neoadjuvant chemotherapy. A complete blood count was carried out at baseline before beginning the neoadjuvant chemotherapy. The absolute count of neutrophils was divided by the absolute count of lymphocytes to calculate the NLR. *Results:* Of the study patients, 18 (39.1%) were considered to have a low NLR (NLR < 1.76), and 28 (60.9%) were considered to have a high NLR (NLR ≥ 1.76). Patients with a low NLR had 18-fold higher rates of pCR when compared to patients with a high NLR (OR 18.1; 95% CI (1.058–310.757); *p* = 0.046). *Conclusions:* Our findings indicate that the pretreatment NLR is a pivotal predictor factor of the pathological complete response in Egyptian breast cancer patients treated with neoadjuvant chemotherapy.

## 1. Introduction

Breast cancer is one of the most widespread cancers affecting women throughout the world. According to the estimates of the American Cancer Society, in 2024, there will be 310,720 new cases of invasive breast cancer in women in the United States and 42,250 deaths [1]. In Egypt, about 32% of all female cancers are attributed to breast cancer. Egyptian women tend to be diagnosed with breast cancer at a younger age, with more than 50% of cases diagnosed in those under 50 years of age [2]. Interestingly, Egypt has approximately double the mortality rate from breast cancer compared to other developing countries (41% versus 23%) [3].

Neoadjuvant chemotherapy is more widely recognized as a breast cancer treatment before definitive cancer surgery [4]. It has become the standard of care for patients with high-risk early-stage or locally advanced breast cancer [5]. Besides its survival outcomes like adjuvant chemotherapy, neoadjuvant chemotherapy can reduce the extent and size of locally advanced tumors, downstage the lymph node status, control micro-metastasis, and assess the sensitivity of the tumor to the treatment regimen, leading to increasing rates of breast-conserving surgery [6].

Neoadjuvant chemotherapy can provide data about cancer prognosis based on the pathological response of the tumor. The total or near total disappearance of the tumor in the resected specimen after neoadjuvant chemotherapy indicates the achievement of a pathological complete response (pCR), which is considered a powerful predictor of survival and good prognosis, especially in more aggressive subtypes like triple-negative or Her-2-positive breast cancers [7].

On the other hand, patients who show no pCR after neoadjuvant chemotherapy have a poor prognosis and need further chemotherapy to improve long-term outcomes [8]. Unfortunately, the pathological evaluation of the resected tumors normally takes about two weeks or longer before the postoperative treatment regimen can be decided. Thus, the identification of more reliable and readily accessible predictive and/or prognostic factors of response to neoadjuvant chemotherapy is needed to give the patients the appropriate treatment in time and identify those patients with a greater risk of recurrence and those who may benefit from further treatment.

Interestingly, systemic inflammation and immunity have a vital role in cancer development, progression, and metastasis [9]. Neutrophils are related to the inflammatory response induced by the tumor, whereas lymphocytes are mostly related to an immune response against the tumor. Thus, the parameters that indicate a systemic background of reduced inflammation and immune system activation can result in a better response to therapy. Consistent with such a role of the immune system, peripheral indicators of inflammation/immunity, including the neutrophil-to-lymphocyte ratio (NLR), which is the ratio between the absolute counts of neutrophils and lymphocytes, have been extensively studied for possible correlations with pCR.

A number of recent studies [10,11,12,13,14] have evaluated the role of NLR as a predictor of response in breast cancers after neoadjuvant chemotherapy. However, the results were controversial, and there were limited data regarding the Egyptian population. Therefore, the current study aimed to explore the association of pretreatment NLR with pCR in a cohort of Egyptian breast cancer patients who received neoadjuvant chemotherapy.

## 2. Materials and Methods

The present study was observational and retrospective and was carried out at a single center in the Clinical Oncology and Nuclear Medicine Department, Menoufia University, Egypt. All participants provided their written informed consent. The study was in agreement with the Declaration of Helsinki [15] and approved by the Ethics Committee of the Faculty of Medicine, Menoufia University, Egypt. The study was registered in ClinicalTrials.gov with ClinicalTrials.gov identifier: NCT06595758. Forty-six (N = 46) breast cancer females were recruited from October 2022 to November 2023. The breast cancer was definitively diagnosed through a core needle biopsy. The patients received preoperative neoadjuvant chemotherapy and then underwent surgery. The most common regimen included anthracycline-based chemotherapy (one cycle every 21 days of the AC regimen containing doxorubicin (60 mg/m^2^) and cyclophosphamide (600 mg/m^2^) for four cycles) and then followed by taxanes. Neoadjuvant trastuzumab was administered to patients whose tumors were Her-2-positive. Surgical procedures involved modified radical mastectomy (MRM) or breast-conserving surgery (BCS), as clinically indicated. The patients included in the study had a performance status of 0 or 1, adequate liver, bone marrow, and kidney functions, and no contraindication to chemotherapy. Exclusion criteria included bilateral breast cancer, pregnancy, lactation, male patients, primary surgery, distant metastases, other malignancies, infections, inflammations, hematological disorders, autoimmune diseases, and patients who are on non-steroidal anti-inflammatory drugs (NSAIDs), and steroidal or antibiotic therapy.

### 2.1. Pathological Assessments

The core needle biopsy specimens were addressed for immunohistochemistry (IHC) staining to assess the status of the Ki-67 proliferation index, human epidermal growth factor 2 (Her-2), progesterone receptor (PR), and estrogen receptor (ER).

When at least one percent of the tumor nuclei was positive for ER or PR, tumors were considered to be positive for these hormone receptors [16]. According to the American Society of Clinical Oncology/College of American Pathologists (ASCO/CAP) guidelines, any tumor with a score of 3+ via IHC or 2+ after a subsequent confirmation with fluorescence in situ hybridization (FISH) was considered to be positive for Her-2 [17].

The cut-off criteria for Ki-67 differ from one center to another. When the proliferation index was ≥14%, we considered a sample to be Ki-67-high, and samples <14% were considered Ki-67-low [18].

All resected tumors were evaluated to determine the pathologic effect of neoadjuvant chemotherapy. The Miller–Payne grading system defined the pCR, where all invasive tumor cells completely disappear in lymph nodes and breast tissue regardless of residual ductal carcinoma existing in situ (DCIS) [19].

### 2.2. Data Collection and Blood Samples

The patient’s medical records were analyzed for the collection of baseline characteristics and clinicopathologic data such as age, histologic type, menopausal status, Ki-67 levels, progesterone receptor (PR) status, human epidermal growth factor 2 (Her-2) status, and estrogen receptor (ER) status.

A complete blood count was carried out at baseline before beginning the neoadjuvant chemotherapy. The absolute count of neutrophils was divided by the absolute count of lymphocytes to calculate the NLR.

### 2.3. Statistical Analysis

The receiver operating characteristic (ROC) curve analysis was carried out to assess the pretreatment NLR cut-off point. Parametric data were displayed as the mean ± SD, whilst categorical data were displayed as frequencies and percentages. To compare the categorical variables, the chi-square test (χ^2^) was used. The predictive power of different variables was evaluated using binary logistic regression, including univariate and multivariate analyses. The odds ratio (OR) was determined with corresponding 95% confidence intervals (95% CI). The value of *p* < 0.05 was found to be statistically significant. For data analysis, the IBM SPSS Statistics ver. 23 was utilized (Chicago, IL, USA).

## 3. Results

### 3.1. Patients’ Characteristics

**Figure 1:**
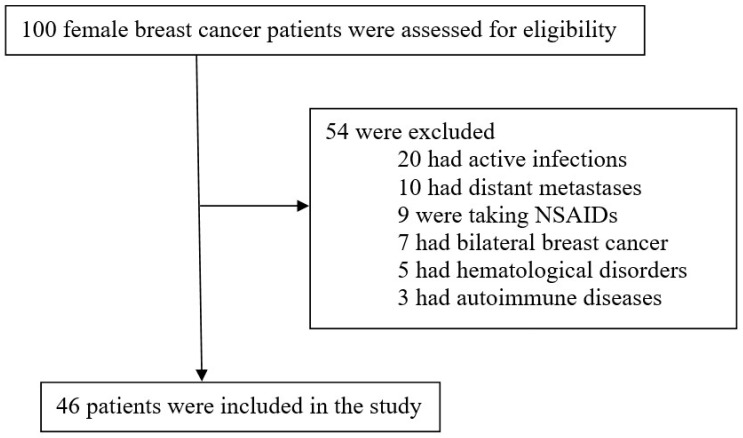
Study flow diagram.

We identified 100 female breast cancer patients treated with neoadjuvant chemotherapy. Only 46 patients fulfilled the study’s inclusion criteria and were enrolled in the study, as demonstrated in Figure 1.

Table 1 shows the patients’ baseline characteristics such as age, menopausal status, histologic type, Her-2 status, PR status, ER status, Ki-67 levels, and NLR values. At diagnosis, the median age was 45.5 years (range 31–68). Of the study patients, 29 (63.0%) were premenopausal, 36 (78.3%) were ER-positive, 34 (73.9%) were PR-positive, 27 (58.7%) were Her-2-positive, 26 (56.5%) exhibited tumors with a high Ki-67 expression (≥14%), 18 (39.1%) had a low pretreatment NLR (<1.76), only 3 (6.5%) had triple-negative breast cancer (TNBC), 44 (95.7%) had invasive ductal carcinoma, which was the prevalent histology, and 5 (10.9%) exhibited tumors with a high grade (GIII).

**Figure 2:**
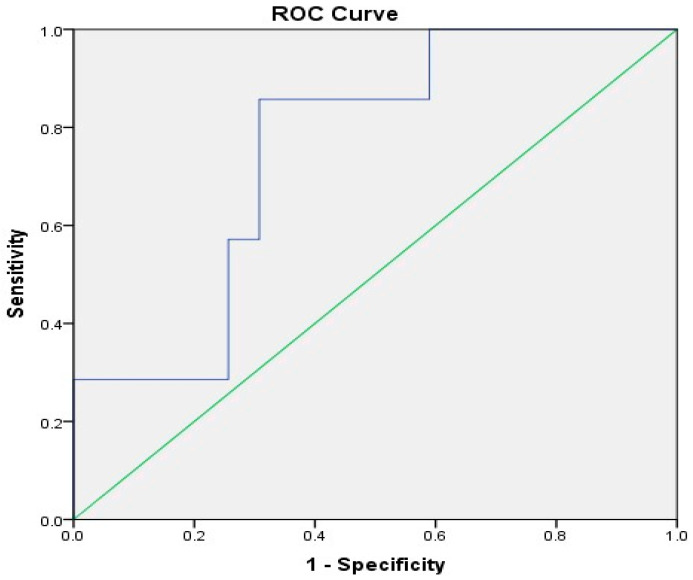
The ROC curve analysis of the baseline NLR.

The ROC curve analysis revealed that the pretreatment NLR cut-off point was 1.76 with a specificity and a sensitivity of 69.2% and 85.7%, respectively, and there was statistical significance (*p* = 0.034; AUC = 0.755, 95% CI: 0.585–0.924), as illustrated in Figure 2. Of the study patients, 18 (39.1%) were considered to have a low NLR (NLR < 1.76), and 28 (60.9%) were considered to have a high NLR (NLR ≥ 1.76).

### 3.2. Relationship Between Baseline Characteristics and pCR

The number of pCR patients was 7 (15.2%), and the number of non-pCR patients was 39 (84.9%) following neoadjuvant chemotherapy, as shown in Table 2. Patients with hormone receptor negativity (HR-), triple-negative subtypes, and low NLR had a significant association with pCR following neoadjuvant chemotherapy, as shown by the univariate analysis in Table 3. In comparison to patients with HR+, patients with ER- and PR- had high rates of pCR (OR 17; 95% CI (2.569–112.479); *p* = 0.0033 and OR 33; 95% CI (3.346–325.478); *p* = 0.0028, respectively). Similarly, patients with a low NLR had higher pCR rates when compared to patients with a high NLR (OR 13.5; 95% CI (1.461–124.744); *p* = 0.0218). Triple-negative subtypes showed significantly higher pCR rates when compared to non-triple negative (NTN) subtypes (OR 15.2; 95% CI (1.157–199.642); *p* = 0.0383). A subsequent multivariate analysis showed that only patients with a low NLR revealed a significant association with pCR. In comparison to patients with a high NLR, patients with a low NLR had 18-fold higher rates of pCR (OR 18.1; 95% CI (1.058–310.757); *p* = 0.046), as illustrated in Table 3.

## 4. Discussion

Despite the presence of some factors predicting the response to neoadjuvant chemotherapy such as tumor size, histological grade or stage, molecular subtype, Ki-67 proliferation index, Her-2 status, PR status, and ER status [20,21,22,23], there is an urgent need for the identification of more reliable and readily accessible predictive and/or prognostic factors of response to neoadjuvant chemotherapy with a high possibility to be carried out in routine clinical practice and consequently, better adjustments for the therapy for each individual patient.

Over the last few years, inflammatory blood markers, particularly the NLR, have been identified as predictive and/or prognostic factors of response to neoadjuvant chemotherapy in breast cancer patients [24,25,26]. Due to the racial differences in breast cancers and the lack of such studies among Egyptian breast cancer patients, the current study was conducted to explore the role of NLR as a predictive factor of pCR in a cohort of Egyptian breast cancer patients treated with neoadjuvant chemotherapy.

The main interesting findings of this study were that the low levels of the pretreatment NLR were significantly related to pCR in both univariate and multivariate analyses when combined with other factors, such as triple-negative subtypes as well as hormone receptor negativity.

We selected an NLR cut-off value of 1.76 in order to differentiate between patients who were and were not likely to achieve pCR following neoadjuvant chemotherapy. This cut-off value was selected based on the ROC curve analysis. The study population was classified according to this cut-off value into groups with a low NLR (NLR < 1.76) and a high NLR (NLR ≥ 1.76). There is currently no unified consensus regarding optimal NLR cut-off values for the evaluation of breast cancer patients. The cut-off values selected in the present study are consistent with previous studies using cut-off values ranging from 1.7 to 4 [27]. On the other hand, Zhu et al. [14] selected an NLR cut-off value of 1.695, which was lower than the values reported in our study and prior studies.

The clinical relevance of the present study is to highlight the importance of precision medicine and the personalized treatment of breast cancer, as the early prediction of responses to neoadjuvant chemotherapy could help in better patient selection and adjustments to (by increasing or decreasing) chemotherapy regimens. Thus, this could decrease the possibility of observing residual disease that is considered to be more resistant than the primary tumor [28]. In addition, the use of neoadjuvant chemotherapy in patients with a lower NLR could achieve higher rates of pCR and improve long-term outcomes.

Chemoresistance is a critical clinical problem in the treatment of breast cancer, especially triple-negative subtypes [29]. For successful chemotherapy, it is important to identify markers that better predict response and, thus, select patients more likely to respond to chemotherapy. Moreover, it is crucial to develop new targets that improve chemosensitivity in the selected patients. For example, the FDA-approved drug pertuzumab combined with docetaxel and trastuzumab as a form of neoadjuvant therapy can significantly improve pCR in patients with Her-2-positive breast cancer [30]. Furthermore, Restivo et al. [31] showed that the use of aspirin in patients with rectal cancer during preoperative chemoradiation was related to the high downstaging of tumors, a good pathologic response, and a slightly higher pCR rate.

In agreement with the present study, Zhu et al. [14] found that the independent predictor of pCR after neoadjuvant chemotherapy is a low baseline NLR. Additionally, Qian et al. [32] showed that a low pretreatment NLR is substantially related to pCR only in the univariate analysis.

A number of studies have shown the important role of the NLR as a predictor of response and a prognostic factor of survival and long-term outcomes. For example, Asano et al. [33] mentioned that triple-negative breast cancer patients with a low NLR show a higher rate of pCR when compared to those with a high NLR and have significantly longer overall survival and disease-free survival only in the univariate analysis, concluding that a low NLR may indicate favorable outcomes and high efficacy in triple-negative breast cancer patients after neoadjuvant chemotherapy. Similarly, Chae et al. [34] revealed that patients with a low NLR show a significantly greater pCR rate and recurrence-free survival than those with a high NLR, suggesting that the NLR in triple-negative breast cancer patients can predict pCR to neoadjuvant chemotherapy and could be a prognostic factor of recurrence. Moreover, Chen et al. [35] stated that the pCR rate is higher in patients with a lower pretreatment NLR than those with a higher NLR, indicating that NLR in breast cancer patients may pivotally predict responses to neoadjuvant chemotherapy. Additionally, they found that patients with a higher pretreatment NLR had a significantly lower breast cancer-specific survival rate and relapse-free survival rate compared to those with a lower NLR.

On the other hand, Bae et al. [36] showed that a high NLR in patients with Her-2 negative breast cancer is significantly related to a low rate of pCR to neoadjuvant chemotherapy and bad survival outcomes. Thus, NLR could be a prognostic factor and predictor of treatment response in those patients. Similarly, von Au et al. [13] found that the NLR in the Luminal B/Her-2 negative and postmenopausal subgroups is substantially higher only for patients who can achieve pCR. However, some studies have not found any significant relation between the pretreatment NLR and pCR in breast cancer patients after receiving neoadjuvant chemotherapy [10,11,37,38].

The retrospective nature of this study was one of its limitations. The general applicability of our findings is limited by the relatively small sample size and the fact that samples were recruited from a single center. In addition, the correlation between NLR and tumor-infiltrating lymphocytes, which reflect the immune system in the tumor microenvironment, was not involved in the present study. Moreover, our study lacked any evaluation of patient survival outcomes. Therefore, future prospective multicenter clinical studies with a bigger sample size are warranted to confirm these preliminary results.

However, our study sheds light on the relationship between chemoresistance and inflammation and the possible utility of inflammatory blood-based markers to help predict outcomes, in combination with other biomarkers, in breast cancer patients. Thus, the pretreatment NLR can be considered a predictive factor for chemoresistance and developing novel targets for enhancing chemosensitivity in selected patients.

In addition, our study has shed light on the potential usefulness of NLR as an indicator of immune system activity against breast cancer instead of just being a marker of systemic inflammation. Moreover, additional future studies could investigate the importance of incorporating anti-inflammatory agents into standard chemotherapy to potentially augment the rate of pCR by shifting the phenotype of NLR from inflammatory to immunogenic.

## 5. Conclusions

Our findings indicate that the pretreatment NLR is a pivotal predictive factor of pCR after neoadjuvant chemotherapy in Egyptian breast cancer patients. The pretreatment NLR is a good peripheral blood marker for response. It is more convenient and robust than other pathological parameters as it can be easily measured in routine clinical practice without the necessity for any invasive procedures or specialized equipment.

## Figures and Tables

**Table 1:** Baseline characteristics of breast cancer patients in the study (N = 46).

Variable	N (%)
Age (years)	
<50	29 (63.0%)
≥50	17 (37.0%)
Family history	
Positive	9 (19.6%)
Negative	37 (80.4%)
Menstrual status	
Premenopausal	29 (63.0%)
Postmenopausal	17 (37.0%)
Tumor site	
Right	20 (43.5%)
Left	26 (56.5%)
Histologic type	
IDC	44 (95.7%)
ILC	2 (4.3%)
Histologic grade	
Grade I–II	41(89.1%)
Grade III	5 (10.9%)
ER	
Positive	36 (78.3%)
Negative	10 (21.7%)
PR	
Positive	34 (73.9%)
Negative	12 (26.1%)
Her-2	
Positive	27 (58.7%)
Negative	19 (41.3%)
Molecular subtype	
Triple negative	3 (6.5%)
Non-triple negative	43 (93.5%)
Ki-67	
Low (<14%)	20 (43.5%)
High (≥14%)	26 (56.5%)
NLR	
Low (<1.76)	18 (39.1%)
High (≥1.76)	28 (60.9%)

IDC: invasive ductal carcinoma; ILC: invasive lobular carcinoma; ER: estrogen receptor; PR: progesterone receptor; Her-2: human epidermal growth factor 2; Ki-67: proliferation index; NLR: neutrophil-to-lymphocyte ratio.

**Table 2:** Relationship between baseline characteristics and pCR.

Variable	pCR (N = 7) N (%)	Non pCR (N = 39) N (%)	*p* Value
Age (years)			
<50	3 (42.9%)	26 (66.7%)	0.2295
≥50	4 (57.1%)	13(33.3%)	
Family history			
Positive	2 (28.6%)	7 (17.9%)	0.5142
Negative	5 (71.4%)	32 (82.1%)	
Menstrual status			
Premenopausal	3 (42.9%)	26 (66.7%)	0.2295
Postmenopausal	4 (57.1%)	13(33.3%)	
Tumor site			
Right	3 (42.9%)	17 (43.6%)	0.9713
Left	4 (57.1%)	22 (56.4%)	
Histologic type			
IDC	6 (85.7%)	38 (97.4%)	0.1614
ILC	1 (14.3%)	1 (2.6%)	
Histologic grade			
Grade I–II	5 (71.4%)	36 (92.3%)	0.1022
Grade III	2 (28.6%)	3 (7.7%)	
ER			
Positive	2 (28.6%)	34 (87.2%)	0.0005 *
Negative	5 (71.4%)	5 (12.8%)	
PR			
Positive	1 (14.3%)	33 (84.6%)	0.0001 *
Negative	6 (85.7%)	6 (15.4%)	
Her-2			
Positive	4 (57.1%)	23 (59.0%)	0.9278
Negative	3 (42.9%)	16 (41.0%)	
Ki-67			
Low (<14%)	1 (14.3%)	19 (48.7%)	0.091
High (≥14%)	6 (85.7%)	20 (51.3%)	
Molecular subtype			
Triple negative	2 (28.6%)	1 (2.6%)	0.01 *
Non-triple negative	5 (71.4%)	38 (97.4%)	
NLR			
Low (<1.76)	6 (85.7%)	12 (30.8%)	0.0061 *
High (≥1.76)	1 (14.3%)	27 (69.2%)	0.0061 *

pCR: pathological compete response; IDC: invasive ductal carcinoma; ILC: invasive lobular carcinoma; ER: estrogen receptor; PR: progesterone receptor; Her-2: human epidermal growth factor 2; Ki-67: proliferation index; NLR: neutrophil-to-lymphocyte ratio; * statistically significant values, *p* < 0.05.

**Table 3:** Univariate and multivariate analyses of the relationship between patients’ characteristics and pCR.

Variable	Univariate		Multivariate	
	OR (95% CI)	*p* Value	OR (95% CI)	*p* Value
ER				
Negative vs. Positive	17 (2.569–112.479)	0.0033 *	4.4 (0.136–142.8)	0.403
PR				
Negative vs. Positive	33 (3.346–325.478)	0.0028 *	10.9 (0.417–285.955)	0.151
Molecular subtype				
Triple Negative vs. NTN	15.2 (1.157–199.642)	0.0383 *	1.6 (0.042–58.87)	0.807
NLR				
Low vs. High	13.5 (1.461–124.744)	0.0218 *	18.1 (1.058–310.757)	0.046 *

ER: estrogen receptor; PR: progesterone receptor; NTN: non-triple negative; NLR: neutrophil-to-lymphocyte ratio; OR: odds ratio; CI: confidence interval; * statistically significant values, *p* < 0.05.

## Data Availability

The data will be available upon reasonable request from the corresponding author.
